# Antimicrobial and anti-biofilm activity of *Hypericum brasiliense* extract and its fractions on *Staphylococcus* of canine origin

**DOI:** 10.1038/s41598-025-00010-9

**Published:** 2025-08-26

**Authors:** Yasmim de M. Assumpção, Lialyz S. P. Andre, Izabel M. Teixeira, Carolina O. da Fonseca, Francisco P. Machado, Tainara A. N. Ribeiro, Renata F. A. Pereira, Daniela Sachs, Leandro M. Rocha, Bruno Penna

**Affiliations:** 1 Gram-Positive Cocos Laboratory, Biomedical Institute, Federal Fluminense University, Niterói, RJ 24210-130 Brazil; 2 Molecular Epidemiology and Biotechnology Laboratory, Biomedical Institute, Federal Fluminense University, Niterói, RJ 24241-002 Brazil; 3 Natural Products Technology Laboratory, Pharmaceutical Technology Department, Federal Fluminense University, Niterói, RJ 24241-002 Brazil; 4 Microbiological Analysis Laboratory, Institute of Physics and Chemistry, Federal University of Itajubá, Itajubá, MG 37500-903 Brazil

**Keywords:** *Staphylococcus pseudintermedius*, *Staphylococcus coagulans*, *Hypericum brasiliense*, Biofilm, Drug screening, Pharmacology, Bacteriology, Biofilms

## Abstract

The growing resistance to antimicrobials, partly due to the ability to form biofilms, poses a challenge for developing new antimicrobial agents. This study assessed the antimicrobial and antibiofilm activity of *Hypericum brasiliense* extract, Japonicin, and Uliginosin-B against clinical isolates of *S. pseudintermedius* and *S. coagulans* from dogs. The minimal inhibitory concentration (MIC) and the minimal bactericidal concentration (MBC) were determined. In vitro antibiofilm activity was evaluated and showed promising results in young (6 h) and mature (24 h) biofilms. *S. pseudintermedius* and *S. coagulans* were highly sensitive to all tested substances. All tested isolates exhibited low MIC and MBC values, particularly for Uliginosin-B. Japonicin demonstrated higher MIC and MBC values. The analysis of antibiofilm activity revealed inhibition of biofilm formation and even disruption of pre-formed biofilms at various concentrations, including sub-inhibitory ones. *H. brasiliense* and its fractions exhibited antimicrobial and antibiofilm activity, offering promising prospects for treating infections caused by biofilm-forming bacteria.

## Introduction

*Hypericum brasiliense*, a native non-endemic species from Brazil, is distributed among the Southern and Southeastern regions^1^. The species demonstrated promising results in killing some Gram-positive and Gram-negative bacteria and destroying their biofilm^2,3^. *H. brasiliense* are chemically composed of flavonoids, xanthones, and phloroglucinols^3,4^. The phloroglucinols Japonicin A, Uliginosin A, Uliginosin B, and Hyperbrasilol A were previously isolated from *H. brasiliense*, and their structures were established by spectroscopic methods, all of which have been proven to have antibacterial effect against *Bacillus subtilis*^4^. Despite its potential, the activity of *H. brasiliense* on staphylococci species, especially of veterinary origin, still needs to be evaluated.

The global epidemic of multidrug-resistant bacteria puts unprecedented pressure on the medical research community. Multidrug-resistant, methicillin-resistant staphylococci, especially methicillin-resistant *Staphylococcus. pseudintermedius* (MRSP), have emerged in small animals worldwide, often limiting treatment options for bacterial skin infections^5^. Additionally, some studies reported cases of zoonotic dog-to-human transmission of *S. pseudintermedius* and even MRSP^6^. Furthermore, high levels of antimicrobial multi-resistance among these isolates make MRSP-associated infections challenging to treat veterinary-licensed systemic antimicrobial agents^7^.

On the other hand, little is known about the diversity and zoonotic potential of *Staphylococcus. coagulans*. However, studies reported that this species causes infection in surgical sites and prosthetic material implanted in humans. This demonstrates that this is also relevant for the zoonotic potential, representing a significant public health concern^8,9^. High-level antimicrobial resistance makes infections difficult to treat with veterinary-licensed systemic antimicrobial agents^7^, which is today a One Health issue considering the potential zoonotic of these microorganisms^10^.

Biofilm formation is a crucial species virulence factor of the *Staphylococcus* genus^11^. Biofilm is an aggregate of bacterial cells surrounded by an extracellular matrix formed by polysaccharides, proteins, and other organic components, which allow them to adhere to biotic or abiotic surfaces^11,12^. This resistance mechanism contributes to these pathogens persisting in the host, even after treatment with antimicrobials. Thus, this study evaluates the potential bactericidal and bacteriostatic activity of *H. brasiliense* extracts and their fractions against *S*. *pseudintermedius* and *S. coagulans* clinical isolates.

## Results

### Biofilm formation

**Fig. 1:**
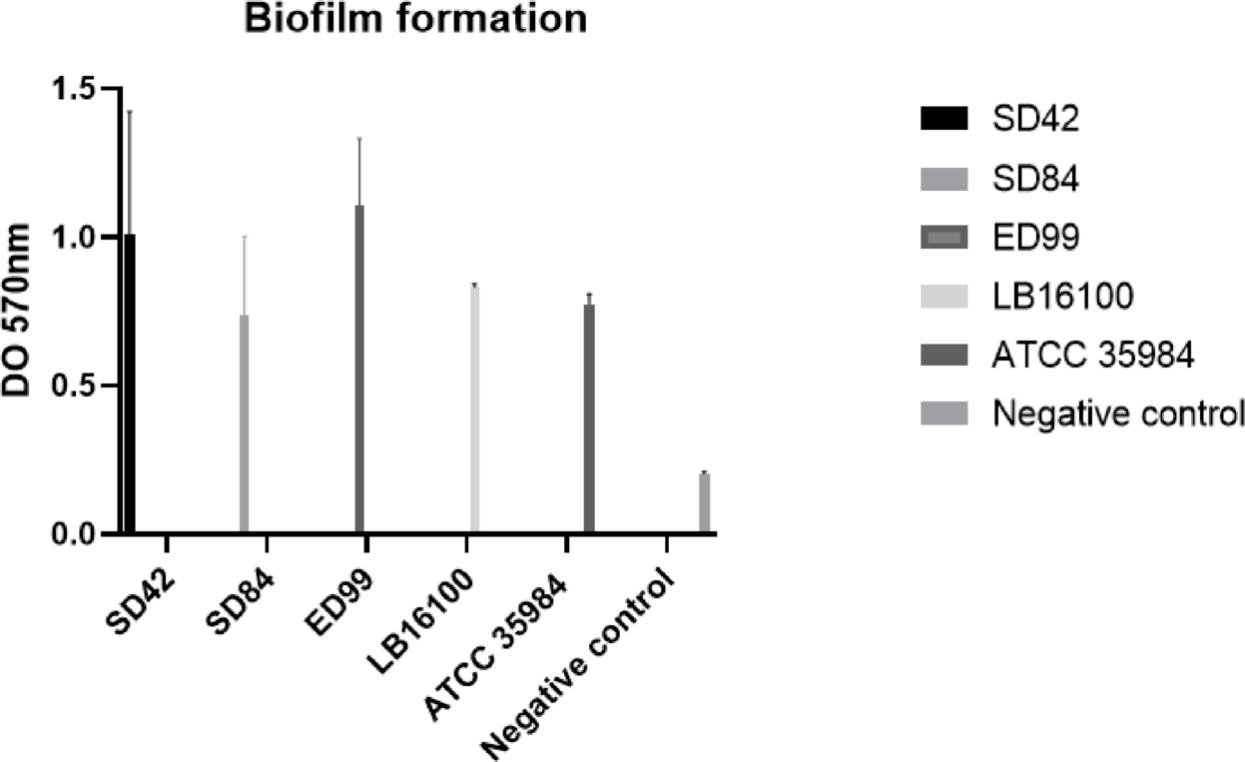
Biofilm formation, by *Staphylococcus coagulans* SD42, SD84; *Staphylococcus pseudintermedius* ED99, LB16100 and *Staphylococcus epidermidis* ATCC35984. The error bars represent the standard deviation of three replicates.

All isolates in the study were considered strong biofilm producers (Fig. 1).

### Samples characterization

We conducted agr typing and searched for virulence genetic determinants among samples analyzed by WGS. Table 1 summarizes the information about the sequenced samples and resistance profile.

**Table 1:** Information regarding carriage of resistance genes, biofilm formation and virulence genes of *S. pseudintermedius* and *S. coagulans* samples sequenced.

ID	Strain	*Agr t*ype	Resistance profile	Beta-lactams	Biofilm formation	Virulence genes
LB16100	MRSP	*Agr IV*	oxa, pen, eno, cip, eri, cli, sut	*blaZ; mecA*	Strong	*hlb, lukS, lukF, siet, se-int*
SD42	MSSS		tob	x	Strong	*sdrC, sdrE, spa, geh, lip, splA, nuc, galE, capsule, hlgB, hlgC, lukS-PV, lukF-PV, vctC, lisR, lgt*
SD84	MRSS		tet	*mecA*	Strong	*efb, fnbA, sdrE, spa, geh, lip, splA, nuc, galE, capsule, hlgB, hlgC, lukS-PV, lukF-PV, lpeA, vctC, lisR, lgt*

### Antibacterial testing

*H. brasiliense* extract and its fractions were used to determine MICs in the study. The MIC value for *H. brasiliense* extract against *S. pseudintermedius* ED99 planktonic cells was 8 μg ml^−1^, while the MBC was 32 μg ml^−1^. Moreover, *S. pseudintermedius* LB16100 planktonic cells were 16 μg ml^−1^, while the MBC was 128 μg ml^−1^. The antimicrobial vancomycin (control) showed MIC and MBC values at 2 and 8 μg ml^−1^, respectively. Regarding *S. coagulans* SD42 and *S. coagulans* SD84 planktonic cells, the MIC value was 32 μg ml while the MBC was 256 μg ml. The MIC value for Uliginosin-B against *S. pseudintermedius* LB16100 planktonic cells was 1 μg ml^−1^, while the MBC was 8 μg ml^−1^. Furthermore, we found MIC of 2 μg ml^−1^ and MBC value of 32 μg ml^−1^ for ED99. The MIC value for Uliginosin-B against *S. pseudintermedius* SD42 and SD84 was 4 μg ml^−1^, while the MBC was 32 μg ml^−1^. About the Japonicin fraction, the values for SD42 and SD84 were 128 μg ml^−1^ for MIC and 256 μg ml^−1^ for MBC. The MIC values for ED99 and LB16100 were 32 µg/mL, and MBC were 256 μg ml^−1^ (Table 2).

**Table 2:** MIC and MBC values for *Hypericum brasiliense* extract, Ulignosin B, Japonicin and Vancomycin against *Staphylococcus pseudintermedius* ED99*, Staphylococcus pseudintermedius* LB16100.

ID	Sample	*Hypericum brasiliense*		Uliginosin B		Japonicin		Van
		MIC	MBC	MIC	MBC	MIC	MBC	MIC
LB16100	*S. pseudintermedius*	16	128	1	8	4	8	4
ED 99	*S. pseudintermedius*	8	32	2	32	32	256	2
SD42	*S. schleiferi coagulans*	32	256	4	32	128	256	4
SD84	*S. schleiferi coagulans*	32	256	4	32	32	256	4

*Staphylococcus coagulans* SD42 and *Staphylococcus coagulans* SD84*.* All results are expreced in μg ml^−1^.

MIC, minimal inhibitory concentration; MBC, minimal bactericidal concentration; Van, vancomycin.

### Effect on biofilm bacterial formation

**Fig. 2:**
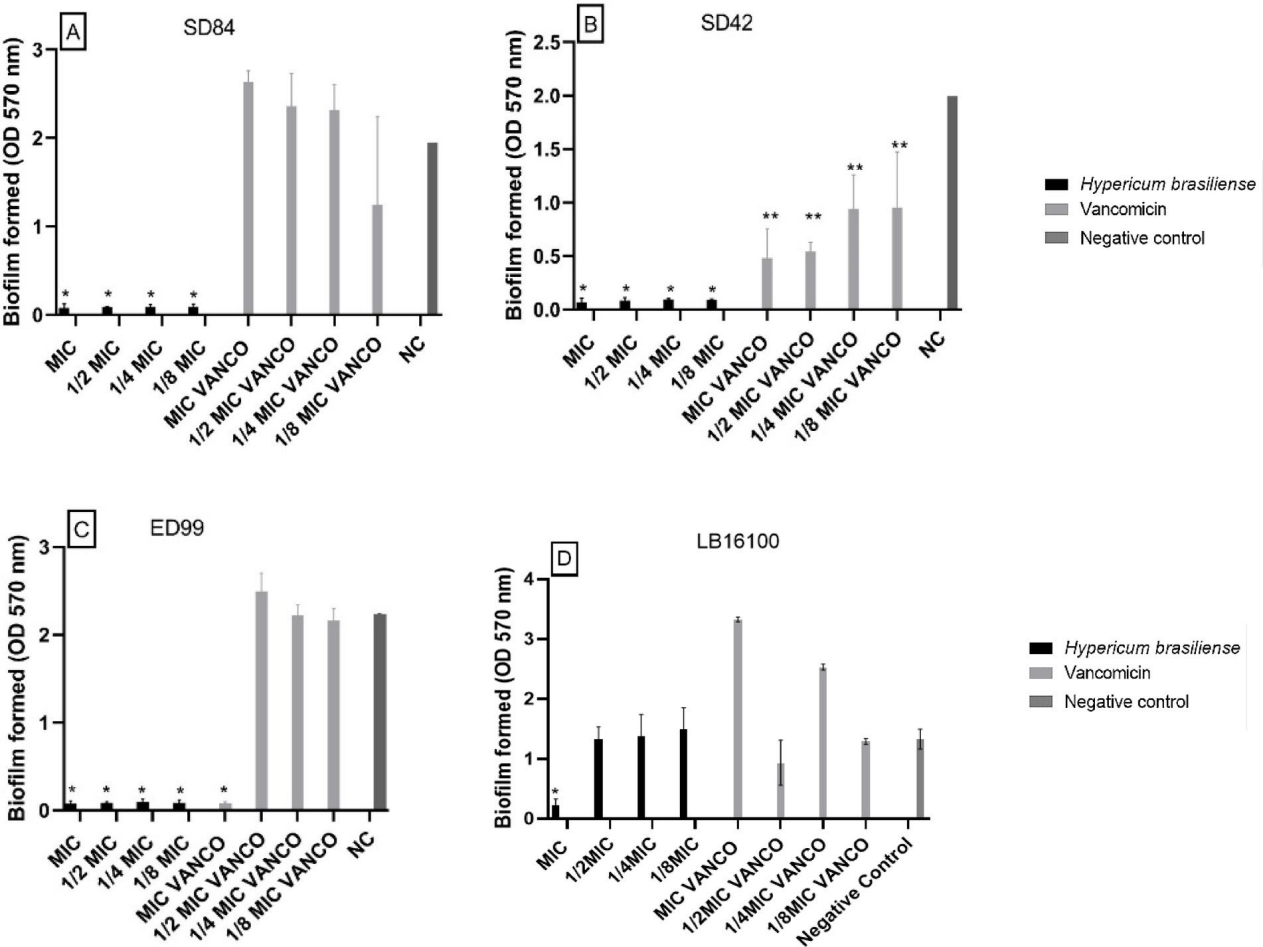
Effect of *Hypericum brasiliense* extract on biofilm formation, by *Staphylococcus coagulans* SD84 (**A**), *Staphylococcus coagulans* SD42 (**B**), *Staphylococcus pseudintermedius* ED99 (**C**) and *Staphylococcus pseudintermedius* LB16100 (**D**). The error bars represent the standard deviation of three replicates. (*, *p* < 0.000001; **, *p* < 0.005).

**Fig. 3:**
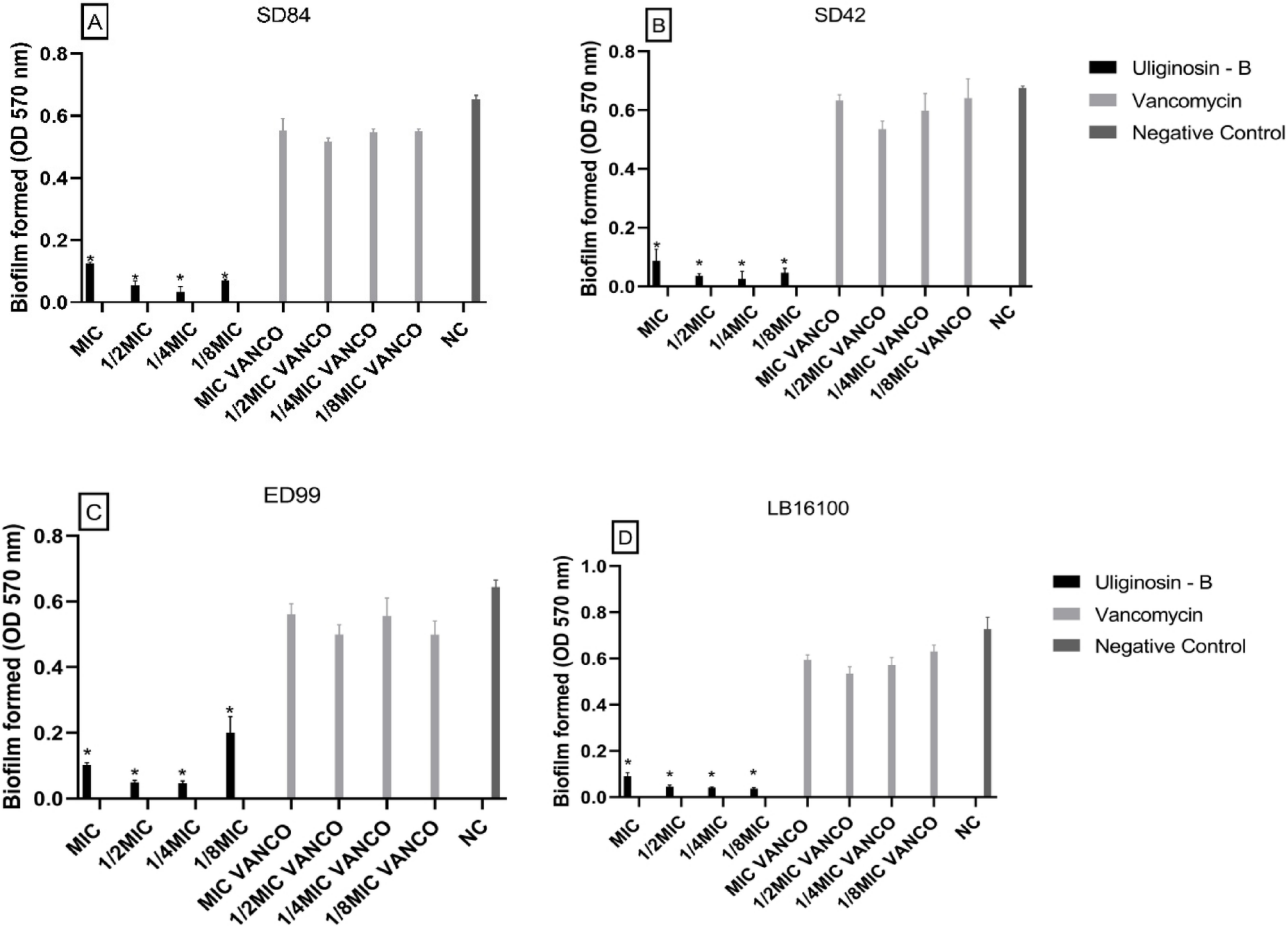
Effect of Uliginosiin-B fraction on biofilm formation by *Staphylococcus coagulans* SD84 (**A**) *Staphylococcus coagulans* SD42 (**B**), *Staphylococcus pseudintermedius* ED99 (**C**) and *Staphylococcus pseudintermedius* LB16100 (**D**). The error bars represent the standard deviation of three replicates. (*, *p* < 0.000001).

**Fig. 4:**
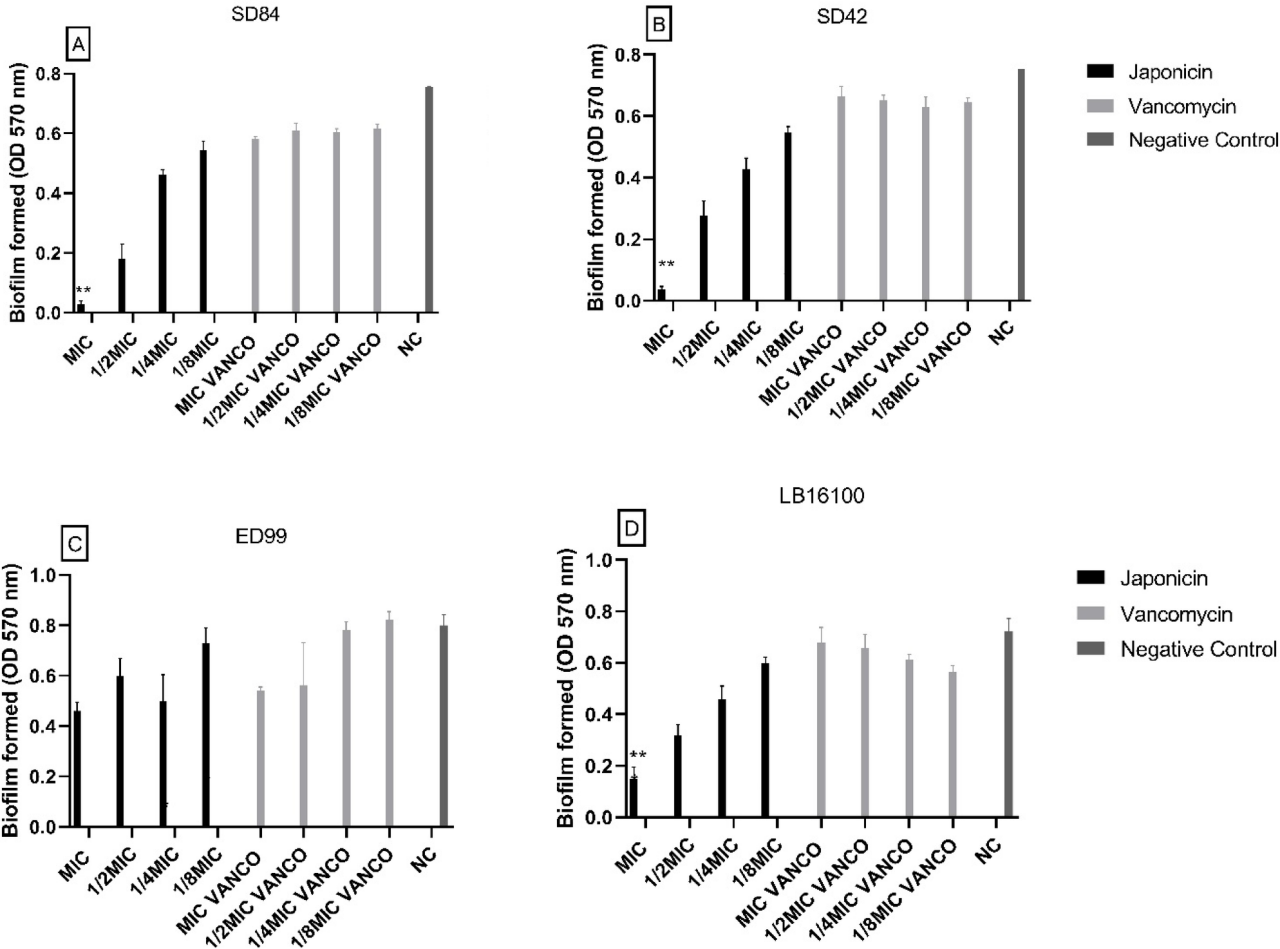
Effect of Japonicin fraction on biofilm formation by *Staphylococcus coagulans* SD84 (**A**) *Staphylococcus coagulans* SD42 (**B**), *Staphylococcus pseudintermedius* ED99 (**C**) and *Staphylococcus pseudintermedius* LB16100 (**D**). The error bars represent the standard deviation of three replicates. (*, *p* < 0.000001; **, *p* < 0.05).

*H. brasiliense* extract significantly inhibited biofilm formation for *S. pseudintermedius* ED99*,* S. *pseudintermedius* LB16100, *S. coagulans* SD42, and *S. coagulans* SD84. The extract of *H. brasiliense* decreased the biofilm production in all tested strains. This could be observed not only in the MIC concentration (90.55%, 85.59% and 94.30% drop of production, respectively) but also in the sub-MICs concentrations. The extract also showed more significant activity against biofilm formation than vancomycin at the same corresponding concentrations for SD84 and ED99, except for a MIC concentration for ED99 (Fig. 2). The biofilm formation was also significantly inhibited in all concentrations tested of Uliginosin-B fraction. The MIC concentration decreased biofilm formation by 84.05% on ED99, 87.44% on LB16100, 87.18% on SD42 and 80.94% on SD84. Japonicin also inhibited biofilm formation more than in MIC concentration (drop of 79.36%) except for ED99 that had no significant reduction (Figs. 3 and 4).

### Effect on pre-formed biofilm

**Fig. 5:**
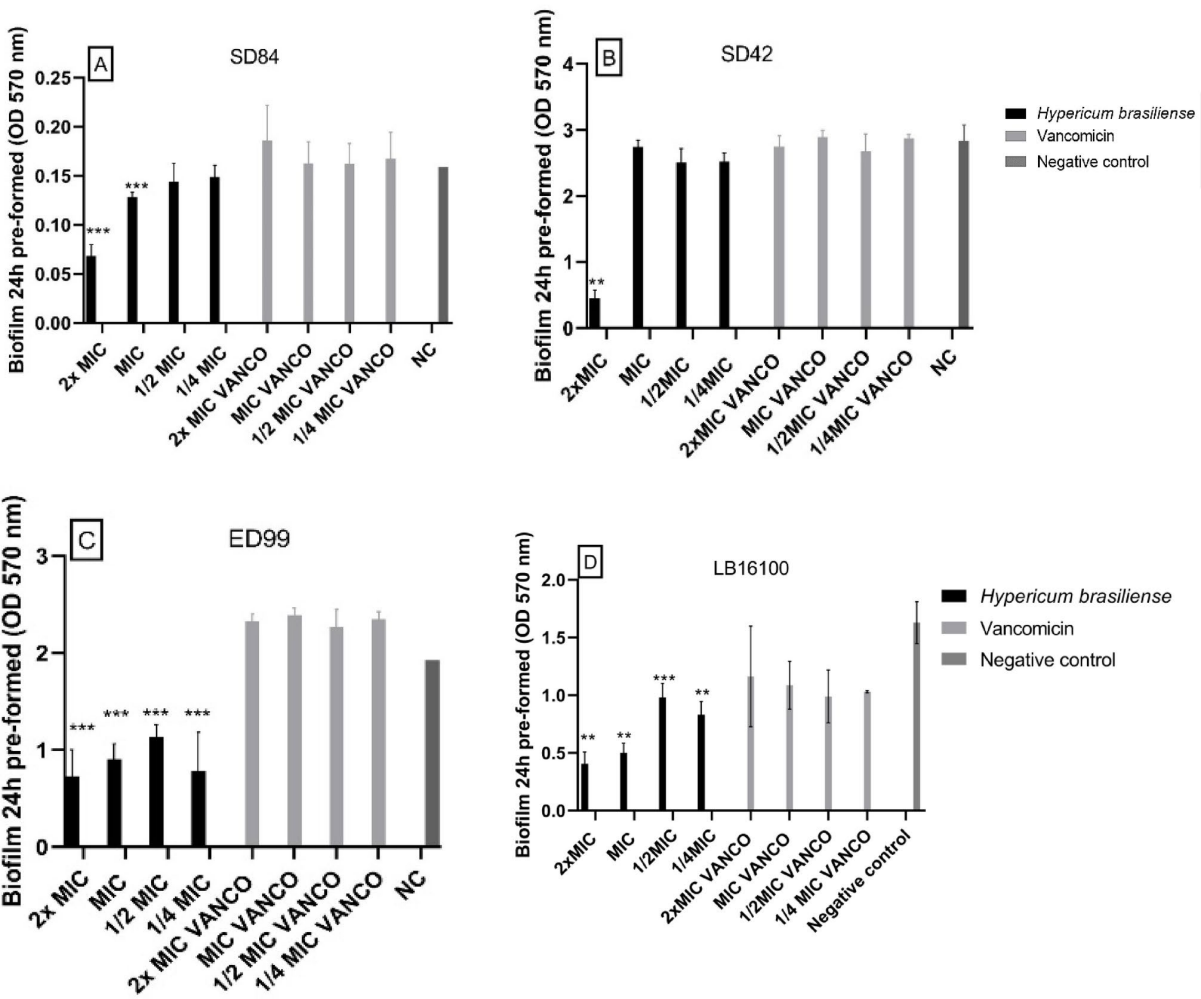
Effect of *Hypericum brasiliense* extract on biofilm 24 h pre-formed by *Staphylococcus coagulans* SD84 (**A**) *Staphylococcus coagulans* SD42 (**B**), *Staphylococcus pseudintermedius* ED99 (**C**) and *Staphylococcus pseudintermedius* LB16100 (**D**). The error bars represent the standard deviation of three replicates. (**, *p* < 0.05; ***, *p* < 0.005).

**Fig. 6:**
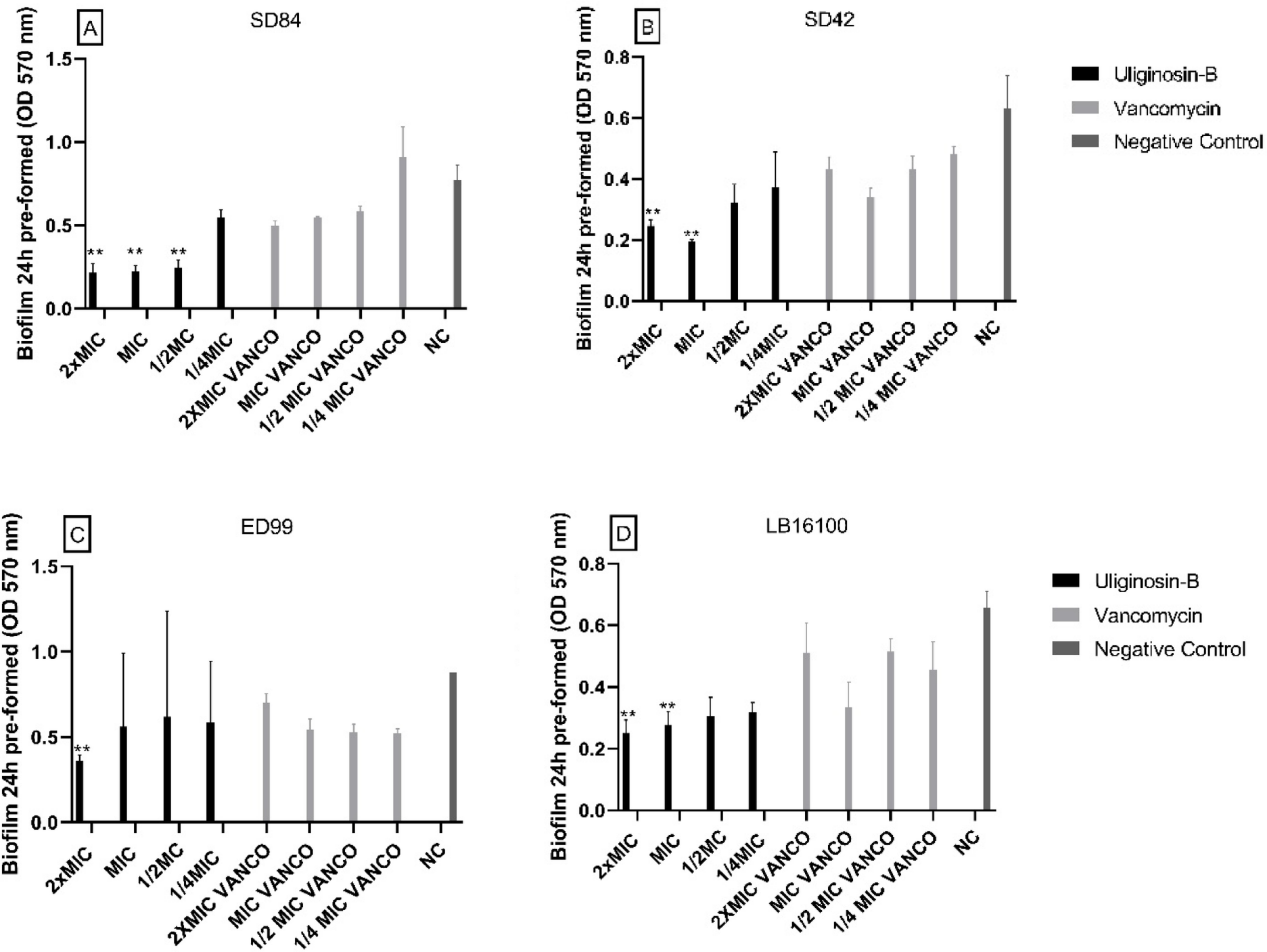
Effect of Uliginosin-B fraction on biofilm 24 h pre-formed by *Staphylococcus coagulans* SD84 (**A**) *Staphylococcus coagulans* SD42 (**B**), *Staphylococcus pseudintermedius* ED99 (**C**) and *Staphylococcus pseudintermedius* LB16100 (**D**). The error bars represent the standard deviation of three replicates. (**, *p* < 0.05).

**Fig. 7:**
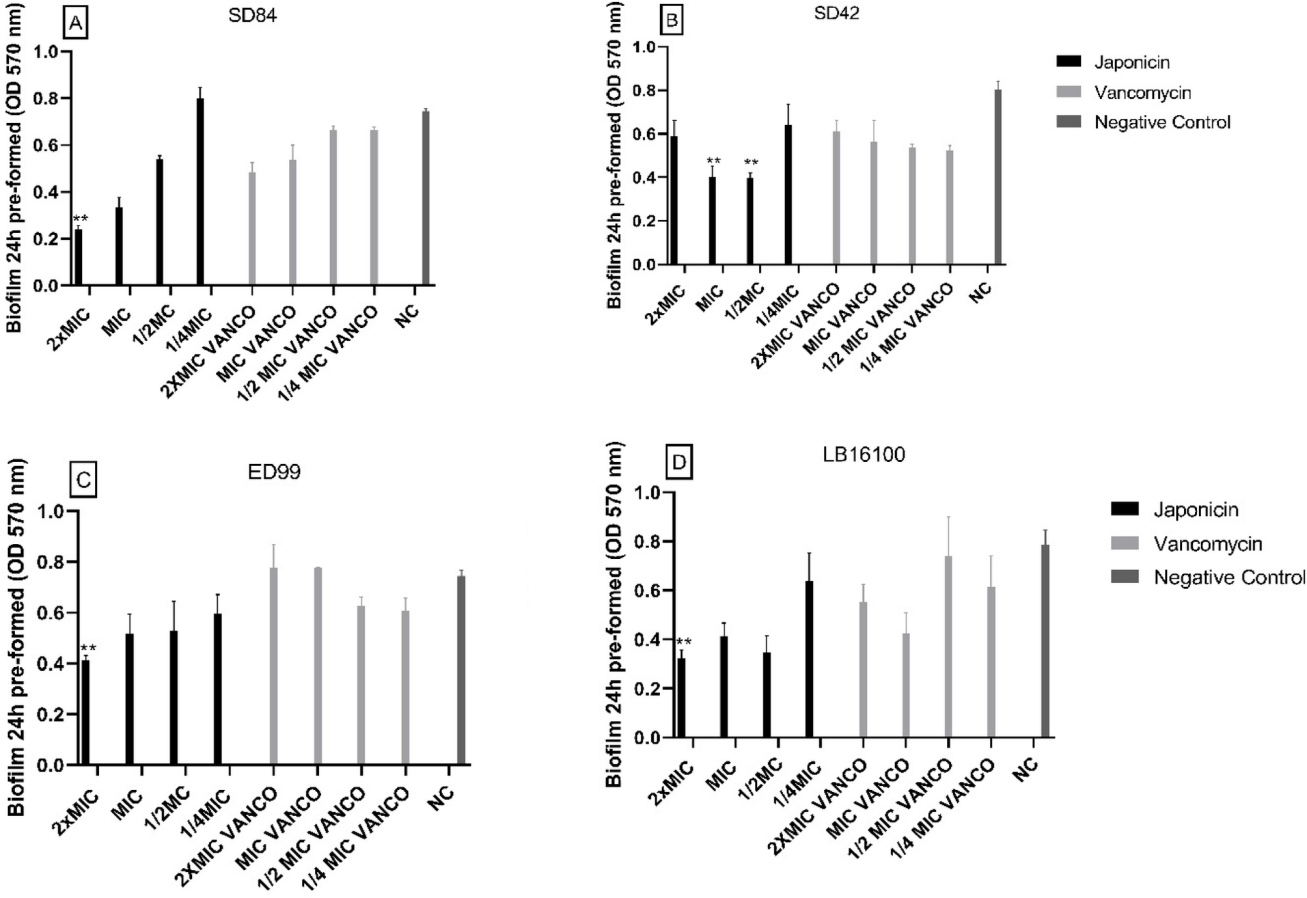
Effect of Japonicin fraction on 24 h pre-formed biofilm by *Staphylococcus coagulans* SD84 (**A**) *Staphylococcus coagulans* SD42 (**B**), *Staphylococcus pseudintermedius* ED99 (**C**) and *Staphylococcus pseudintermedius* LB16100 (**D**). The error bars represent the standard deviation of three replicates. (**, *p* < 0.05).

The pre-formed biofilm experimental results indicated that *H. brasiliense* has good potential for disrupting pre-formed biofilms of *S. pseudintermedius and S. coagulans.* The extract significantly disrupted the pre-formed biofilms at all concentrations tested (2 × MIC, MIC,1/2 × MIC, and 1/4 × MIC) for ED99 (drop of 50.76%, 49.69%, 51.10% and 50.59%, respectively) and LB16100 (drop of 75.10%, 69.42%, 69.71% and 48.99%, respectively) relative to the control (samples without the extract) (Fig. 5). Surprisingly, vancomycin at the corresponding concentrations stimulated biofilm formation (decrease of more than 30.46%) (Fig. 5). In the other hand, the Uliginosin B and Japonicin disrupted the pre-formed biofilms significantly at 2 × MIC concentration for ED99 (59.26% and 44.60%), LB16100 (61.85% and 59.01%) and SD84 (72.1% and 68.%) except for SD42 (Figs. 6 and 7).

## Discussion

As far as we know, this is the first study on *H. brasiliense* extract, Uliginosin- B, and Japonicin activity against clinical *S. pseudintermedius* and *S. coagulans*. In our study, despite being samples with a diverse virulence and resistance profile, the *H. brasiliense* extract showed bacteriostatic and bactericide activity for the clinical isolate of *S. pseudintermedius* and *Staphylococcus coagulans.* In the same way activity against clinical samples of *S. pseudintermedius* and *Staphylococcus coagulans* were observed when they were subjected to inhibitory concentrations of the fractions Uliginosin- B and Japonicin. This reinforces the possibility that the extract and its fractions could be a viable alternative against multiresistant Staphylococci isolates of different origins.

Previous studies performed by França^13^ described the antibacterial effects of the hexane extract of *H. brasiliense* in *S. aureus*, *S. epidermidis*, *S. simulans,* and *S. haemolyticus*. Furthermore, the author described the MIC values of three isolates, Japonicin A (50-25 μg ml^−1^), Uliginosin B (1.5–3.0 μg ml^−1^), and Uliginosin B (3.0 μg ml^−1^) in *S. aureus* and *S. epidermidis*, respectively. Those results suggest that these components in *H. brasiliense* play a crucial role in bioactivity^13^. More recently, Pereira^14^ described the effects of the hexane extract of *H. brasiliense* on an MRSA HU25 strain with 4 μg ml^−1^ MBC value with inhibition of biofilm formation (1/2 × MIC)^14^.

According to Demgne and co-workers^15^, the antibacterial activity of plant extracts is promising for MIC values < 100 μg ml^−1^. In this context, the hexane extract of *H. brasiliense* and its fractions presented very promising results against *S. pseudintermedius*, and *S. coagulans* strains.

In addition to *H. brasiliense*, other species have been studied for their potential antibacterial activity^16^. The most reported *Hypericum* genus is *Hypericum perforatum,* described with an antidepressant, antitumor, analgesic, and pronounced antibacterial properties in Staphylococci, including MRSA strains^17,18^. Those bioactivities are related to flavonoids, phloroglucinols, naphtodianthrones, phenylpropanes, and other constituents^17,18^. Regarding antibacterial potential, several studies described inhibition by *H. perforatum* extracts and suggested that Hyperforin and Hypericin are the main active substances^17,19^. *Hypericum lydium*, for example, demonstrated antibacterial and antivirulence activity against MRSA samples (MIC 16–32 μg ml^−1^)^20^. Furthermore, bark extract from *Hypericum roeperianum* exhibited inhibition in Gram-negative bacteria (64–128 μg ml^−1^) whereas the isolate 8,8-bis (Dihydroconiferyl) diferulate displayed prominent inhibition values (0.5–2 μg ml^−1^). However, it did not show activity in *S. aureus* strains (MIC > 1024 μg ml^−1^)^15^. Growing numbers of staphylococcal infections resistant to available antimicrobials reinforce and corroborate the importance of studies that propose investigating new therapeutic targets, like this study. The biofilm formation is one of the main mechanisms of aggression found in *Staphylococcus* spp.^21^. Furthermore, biofilm production contributes to the persistence of bacteria at the infection site, making treatment with antimicrobials more difficult^22,23^. Pereira^14^ investigated the activity of *H. brasiliense* extract against biofilm forms of *Staphylococcus aureus* HU25 and concluded that this compound significantly reduced biofilm formation in *S. aureus* HU25, corroborating this study’s findings.

It was possible to observe that *H. brasiliense* extract and its fractions (Uliginosin – B and Japonicin) inhibited biofilm formation in inhibitory and subinhibitory concentrations in samples with several virulence factors and high levels of resistance to antimicrobials (ED99, LB 16100, SD42, and SD84). Scanning Electronic Microscopy demonstrated that at 1/2 × MIC concentration, the extract could affect *S. pseudintermedius* and *Staphylococcus coagulans* biofilm formation by preventing aggregation. Comparison with vancomycin, also in inhibitory and subinhibitory concentrations, suggests that the action did not happen only by inhibiting growth or even killing planktonic bacteria (the MIC of vancomycin is smaller and more efficient). It may have prevented cell-to-cell aggregation or even substrate adhesion, thus decreasing the possibility of these communities forming.

We observed the extract effect in mature biofilm in all concentrations for ED99 and LB16100 and 2 × MIC and MIC for SD42 and SD84. This effect may hinder cell aggregation, deranging the biofilm. In the same way, we observed the fraction Uliginosin—B effect in mature biofilm in 2 × MIC and MIC for SD84, ED99 and LB16100 and 2 × MIC for ed99. About Japonicin fraction the action was observed in 2 × MIC for all samples except SD42 (only inhibited in MIC and 1/2 × MIC concentrations). We could be observed that *H.brasiliense* was more effective in the eradication of biofilm formed of theses samples. One of the hypotheses is that it is possible because the extract was more effective due to the presence of both fractions. In this context the extract fractions may act synergistically, but possible antagonistic activities cannot be ruled out. These hypotheses justify the analysis of the crude extract and the fractions as a starting point for more specific studies.

On the other hand, we only observed an effect with vancomycin at the concentration equivalent to twice the MIC in the young biofilm for ED99, and all concentrations were only for SD42. The explanation may be that, at this stage, we still observe planktonic bacteria in the aggregation process, enabling the action of this antibiotic.

## Conclusions

In conclusion, the notable activity of *Hypericum brasiliense* and its fraction Uliginosin-b and Japonicin against *S. pseudintermedius* and *Staphylococcus coagulans* in clinical isolates indicates that it is a promising antimicrobial agent candidate. This is the first study to show the in vitro antibacterial activity of *H. brasiliense* extract and its fractions against the canine skin isolate of *S. pseudintermedius* and *Staphylococcus coagulans.* The study also provided information on MIC and antibiofilm effects regarding using *Hypericum brasiliense* extract and its fractions as an antibacterial agent against *S. pseudintermedius and Staphylococcus coagulans.* The *Hypericum brasiliense* and its fractions mechanism of action is still unclear, and further research is needed to explore this in more detail. Therefore, continued research on *H.brasiliense* and its fractions Uliginosin-B and Japonicin will generate novel ideas for the treatment of diseases caused by *Staphylococcus* sp*.* in the future.

## Materials and methods

### Biofilm formation

Biofilm formation was assessed as recommended by Stepanovic et al.^24^. Biofilm producers were classified as strong, moderate, or weak. A negative control consisted of three wells containing only 200 µl of glucose-supplemented Soy Tryptone Broth (1%), and a positive control consisted of a known strong biofilm-producing *Staphylococcus epidermidis* strain (ATCC 35984) (Fig. 1).

### Sample characterization

*S. pseudintermedius* LB16100, *S. coagulans* SD42, and S. coagulans SD84 strains were selected for whole-genome sequencing and analyzed for antimicrobial resistance using the disk diffusion method, following the recommendations of the Clinical and Laboratory Standards Institute (CLSI), including for animal samples (CLSI-VET, 2013; CLSI, 2017) in previous studies. Among samples analyzed by WGS, we conducted agr typing and a search of virulence genetic determinants. Table 1 summarizes the information about the sequenced samples and resistance profile.

### Bacterial isolates

The strain *S. pseudintermedius* ED99 provided by Dr. Ross Fitzgerald^25^, *S. pseudintermedius* LB16100, *S. coagulans* SD42, and *S. coagulans* SD84 used in this study belonged to the Laboratory of Gram-positive cocci collection from the Biomedical Institute, Universidade Federal Fluminense – UFF.

### Plant material

The *Hypericum brasiliense* was collected in *Trajano de Moraes* city (22° 12′ 17″ S, 43° 11″ 35″ W) on 11/01/2020. A voucher for this specimen was deposited in the Herbarium at the “*Faculdade de Formação de Professores*” – FFP (UERJ), *São Gonçalo*, Brazil, under identification number RB00819157. Authorization to access the botanical material was given by the Sisgen number A314288 The whole plant material (480 g) was dried with forced ventilation in an oven at 40 °C for 48 h. Then, the dried plant material was crushed in a hammer mill and extracted via maceration with hexane at room temperature. The crude extract was obtained after filtration and concentration in a rotary evaporator. Then, the crude extract was redissolved in acetone and filtered to remove fatty acids and other less polar substances. After that, it was filtered and evaporated under reduced pressure to obtain the hexane extract enriched in phloroglucinols (32 g)^4,13^.

### Determination of MIC

After 24 h incubation at 37 °C, two colonies of *S. pseudintermedius* ED99, two of S. *pseudintermedius* LB16100, two of *S. coagulans* SD42, and two of *S. coagulans* SD84 from trypticase soy agar medium (TSA) plate were transferred into Brain Heart Infusion medium (BHI) (5 ml), followed by shaking incubation at 37 °C for 24 h. The suspension was adjusted to 0.5 McFarland standard turbidity. The extract and its fractions were diluted in a 10% dimethyl sulfoxide (DMSO) and applied in a 96-well plate. Serial dilution was performed, reaching concentrations between 256 and 0.5 μg ml^−1^, following the application of bacterial suspension. The plate was incubated at 37 °C for 24 h and dyed with 20 µl of 0.01% resazurin. The MIC was the lowest concentration that the extract and its fractions visualy inhibited the growth of bacterial culture (CLSI 2015). We used vancomycin at concentrations between 8 and 0.0625 μg ml^−1^ as positive control^14^.

### Determination of MBC

For MBC, 1 μl of each serial dilution obtained at the MIC wells was removed after the incubation period and before adding the Resazurin solution. Spots were sown in a petri dish with TSA and incubated at 37 °C for 24 h, followed by observing the presence or absence of bacterial growth. The spot with no bacterial growth was the value of the MBC^14^.

### Effect of the *H. brasiliense* extract and its fractions on biofilm formation

Two colonies of *S. pseudintermedius* ED99, two of *S. pseudintermedius* LB16100, two colonies of *S. coagulans* SD42, and two of *S. coagulans* SD84 were suspended in BHI with added 1% glucose and incubated at 37 °C in a shaker at 150 g. After 20 h of incubation, the inoculum was diluted at 1:100 in BHI with 1% glucose. We applied 200 µl of each dilution corresponding to the specific sub-inhibitory concentrations of the extract (MIC,1/2 × MIC, 1/4 × MIC, and 1/8 × MIC). The applied negative and positive controls contained medium DMSO with S. *pseudintermedius* or *S. coagulans* and medium-DMSO with S. *pseudintermedius* or *S. coagulans* plus vancomycin (MIC,1/2 × MIC, 1/4 × MIC, and 1/8 × MIC), respectively. After 24 h at 37 °C, we measured in a spectrophotometry reader (570 nm) to follow up on the bacterial growth (reading 1). The plate was washed with distilled water and maintained at 70 °C until dry. 200 µl of violet crystal staining was added to each well, followed by 1 min/RT incubation to measure biofilm formation. Then, the staining solution was discarded, the plates dried at 60 °C, and the optical density (OD) was measured at 570 nm (reading 2)^14^.

### Effect of H. brasiliense extract and its fractions on pre-formed biofilms

Sterile 96-well polystyrene plates were filled with 200 µL of the bacterial inoculum 1:100 in BHI with 1% glucose. We removed the supernatant after 24 h (mature biofilm) incubation at 37 °C. The extract of *H. brasiliense* was diluted to 2 × MIC; MIC, 1/2 × MIC, and 1/4 × MIC were applied in triplicate and incubated at 37 °C for 24 h. After that, we washed the plate, dyed it with crystal violet, and measured the absorbance as previously described^26^. Vancomycin at 2 × MIC, MIC, 1/2 × MIC, and 1/4 × MIC were used with and without inoculum as a control^14^.

### Statistical analysis

All the experiments were performed in triplicate and repeated three times. We performed statistical analyses using GraphPad PRISM software (ver. 8). A Student’s t-test analyzed the data and defined statistical significance at *P* < 0.05.

## Data Availability

The complete genome sequences of all isolates used in this study were deposited at GenBank under accession number PRJNA932781.
